# Effectiveness and Outcomes of Noninvasive Positive Pressure Ventilation in Patients With Acute Exacerbations of Chronic Obstructive Pulmonary Disease

**DOI:** 10.7759/cureus.62746

**Published:** 2024-06-20

**Authors:** Mahesh Gudelli, Swetha K, Praisy Thomas Kalathil, Omkar Pimple, Afreen Shahid, Nycy Chandradas, Prerit Sharma, Gangadhar Reddy Mallu

**Affiliations:** 1 Department of Pulmonary Medicine, Krishna Institute of Medical Sciences (KIMS) Hospitals, Secunderabad, IND; 2 Department of General Medicine, Government Medical College, Mahabubnagar, IND; 3 Department of General Medicine, Government Medical College, Kannur, IND; 4 Department of General Medicine, Krishna Institute of Medical Sciences (KIMS), Karad, IND; 5 Department of General Medicine, Dr. B. R. Ambedkar Medical College, Bangalore, IND; 6 Department of General Medicine, Rajarajeshwari Medical College and Hospital, Bangalore, IND; 7 Department of General Medicine, University College of Medical Sciences, New Delhi, IND; 8 Department of Pulmonary Medicine, Yashoda Hospitals, Secunderabad, IND

**Keywords:** dyspnoea, heart rate, respiratory rate, arterial blood gas, non-invasive positive pressure ventilation, chronic obstructive pulmonary disease

## Abstract

Background: Endotracheal intubation and mechanical ventilation in individuals experiencing acute exacerbations of chronic obstructive pulmonary disease (COPD) are associated with several complications. Therefore, utilizing noninvasive positive pressure ventilation (NIPPV) is the suggested initial management for these individuals. The current study was done to assess and compare the clinical and physiological parameters before and after the application of NIPPV and also to evaluate the outcomes of NIPPV.

Methodology: A prospective observational study was conducted on 50 patients with COPD experiencing acute exacerbations. These patients were treated with NIPPV. Measurements of blood pressure, respiratory rate (RR), heart rate (HR), dyspnea using the modified Borg scale, and arterial blood gas (ABG) parameters (pH, PaCO2, and PaO2) were recorded at baseline, one hour, six hours, 24 hours, and daily until discharge. The study's outcomes included the subjects who successfully underwent NIPPV and failed during NIPPV.

Results: NIPPV effectively reduced the dyspnea score from 7.24 ± 1.58 at baseline to 5.53 ± 1.82 at one hour, 4.11 ± 1.75 at six hours, 2.60 ± 1.03 at 24 hours, and 1.26 ± 0.44 at the time of discharge. Significant improvements were also observed in HR and RR (*P* < 0.001). When compared to the baseline, the pH level was significantly maintained, PaCO2 was decreased, and PaO2 was increased at various times. Mortality was observed in four patients.

Conclusions: NIPPV was successful in 42 (84%) patients, with improvements in ABG and pH for early recovery and reduced hospital stay.

## Introduction

Chronic obstructive pulmonary disease (COPD) is the most common lung disorder with paramount disability and imposes significant morbidity and mortality [1]. Previous reports show that according to the global disease mortality rate, by the end of 2030, COPD might occupy the third position [2]. COPD also causes a marked economic burden due to increased treatment costs and also affects the quality of life [3]. Among the various respiratory diseases, COPD is the primary cause of disability and the second most significant contributor to disability-adjusted life years (DALYs) during the year 2016 [4]. In 2016, around 32% of global DALYs as a result of COPD were observed in India, and it is also responsible for 75.6% of the total DALYs associated with chronic respiratory disorders in India [4]. The current treatment scenario for managing COPD with reduced respiratory function includes antibiotics, bronchodilators, and maintenance of water-electrolyte balance. While these strategies can improve the patient’s condition, they do not typically lead to significant improvements in clinical symptoms. The clinical utility of invasive mechanical ventilation usually enhances patient outcomes and also reduces morbidity and mortality. Typically, the procedure involves creating an artificial airway by inserting a catheter into the patient's throat and thus preventing the obstructive airway. However, invasive mechanical ventilation imposes certain side effects such as lung damage as a result of aspiration and ventilator-associated pneumonia, affecting the treatment outcome [5]. The noninvasive positive pressure ventilation (NIPPV) method has recently gained much attention due to the patient’s compatibility. NIPPV is usually performed by an oral-nasal mask, nasal or complete mask, or helmet attached to the ventilator and thus restricts the requirement of an artificial airway and reduces damage to the upper respiratory tract [6]. NIPPV is widely used for various subsets of patients affected with neonatal respiratory distress syndrome, premature infants, muscular dystrophy, and in certain patients where it can reduce bronchopulmonary dysplasia and also the need for tracheal intubation ventilation [7,8]. Against this backdrop, the present study aimed to examine and compare the clinical and physiological parameters before and after the use of NIPPV in subjects with acute exacerbation of COPD. Additionally, the study aimed to determine the outcome of NIPPV in these patients.

## Materials and methods

The present research was a prospective, observational study conducted on 50 COPD patients with acute exacerbation in the Department of Pulmonary Medicine, Yashoda Hospital, Secunderabad, and this protocol was approved by the Institutional Ethics Committee at Yashoda Group of Hospitals, Hyderabad, India (IEC-YAMER/DNBT-06/2017). This study was reported according to the Strengthening the Reporting of Observational Studies in Epidemiology (STROBE) statement for observational studies.

Sample size estimation

A power analysis was conducted using the dependent sample t-test by G*Power software (Heinrich-Heine-Universität Düsseldorf, Düsseldorf, Germany) to fix a sample size with the help of an alpha of 0.05, a power of 0.80, a medium effect size (dz = 0.5), and two tails [9]. Therefore, the obtained sample size was 34 as per the above calculation, while for the current study, a sample size of 50 was considered for analysis.

Inclusion criteria

Subjects with moderate to severe dyspnea lasting less than two weeks, in addition to meeting any one of the following criteria, were included in the study: respiratory rate (RR) >25/minute, clinical evidence of elevated breathing workload, and pH < 7.35-7.25.

Participants with a partial pressure of carbon dioxide (PaCO2) exceeding 45 mmHg after aggressive medical treatment, including oxygen supplementation, were included in the study. Additionally, patients with patent airways and minimal secretions were also included.

Exclusion criteria

Patients with cardiac failure, upper gastrointestinal (GI) bleeding, alteration in hemodynamics, undrained pneumothorax, unstable arrhythmias, neuropathy, myocardial infarction, dental occlusion, facial trauma, and upper airway obstruction were excluded from the study.

Study procedure

Before commencing the procedure, the patients or their caregivers were provided with written information and obtained their consent. Baseline clinical evaluations, such as the clinical history of the patients, were recorded. Respiratory evaluations such as Borg dyspnea score, RR, heart rate (HR), and arterial blood gas (ABG) analysis were also done. Blood investigations and chest X-rays were also performed. In this study, the BiPAP A-40 series ventilator support system (Respironics, Inc., Murrysville, PA) was used to administer NIPPV to the patients. The head end of the bed was inclined at a 45º angle. The patients were subjected to inspiratory positive airway pressure (IPAP) of 10 cm H_2_O and expiratory positive airway pressure (EPAP) of 4-5 cm H_2_O, respectively. IPAP was subjected to a gradual increase at the rate of 5 cm H_2_O for a 10-minute gap to reach a pressure target of 20 cm H_2_O until a desired therapeutic response was achieved. Supplemental oxygen therapy was done using NIPPV till a target oxygen saturation of 88%-92% was achieved.

Subsequently, after treatment initiation, the patients were monitored for the first hour. Any discomfort to the patients or the mask intolerance was also observed. After the continuous application of NIPPV, there was an intermittent termination of drinking and eating. Standard administration of bronchodilators, intravenous (IV) hydrocortisone, and antibiotics was performed along with NIPPV. Blood pressure, RR, HR, and dyspnea using a modified Borg scale were recorded at the baseline, one hour, six hours, 24 hours, and daily until discharge. Patients who exhibited deterioration, as evidenced by elevated PaCO2 or alterations in pH, a Glasgow Coma Scale score of less than 8, hemodynamic changes with copious secretions, or an inability to tolerate the facemask, were intubated and recorded as experiencing NIPPV failure. Weaning from NIPPV was recommended based on clinical improvements such as a reduction in RR to less than 24/minute, HR to less than 100/minute, normalization of pH, PaCO2 less than 55 mmHg, and oxygen saturation levels greater than 90% during ABG analysis.

The NIPPV outcome was estimated in terms of subjects cured by NIPPV and failed during NIPV. Recorded clinical variables included dyspnea score, RR, HR, ABG parameters (pH, PaCO2, and PaO2), NIPPV, hospital stay duration, and potential complications.

Statistical analysis

The data were represented as mean ± standard deviation. The comparison of variables at various time intervals was done using the one-way analysis of variance (ANOVA) test. A *P*-value < 0.05 was considered as statistically significant. Analysis was conducted using SPSS version 25.0 (IBM Corp., Armonk, NY).

## Results

The demographic and clinical characteristics of the participants are shown in Table 1.

**Table 1:** Demographics and baseline clinical variables of the study participants. SD, standard deviation; bpm, beats per minute; PaCO2, partial pressure of carbon dioxide; PaO2, partial pressure of oxygen

Parameters	Values
Age (mean ± SD) (years)	65.92 ± 8.38
Gender	
Male (*n*, %)	42 (84%)
Females (*n*, %)	8 (16%)
Smoking history	
Yes (*n*, %)	43 (86%)
No (*n*, %)	7 (14%)
Smoking (pack years) (*n*, %)	
≤10	2 (4%)
11-20	24 (48%)
21-30	15 (30%)
≥31	2 (4%)
Dyspnea score (mean ± SD)	7.24 ± 1.58
Respiratory rate (minutes) (mean ± SD)	33.86 ± 4.98
Heart rate (bpm) (mean ± SD)	105.42 ± 10.28
pH (mean ± SD)	7.24 ± 0.08
PaCO2 (mean ± SD)	75.68 ± 15.14
PaO2 (mean ± SD)	57.4 ± 13.17

Out of the 50 patients who received NIPPV, 6 (12%) deteriorated and required intubation within six hours. The remaining 44 patients continued to receive NIPPV beyond six hours. Additionally, two patients (4%) left against medical advice.

In this study, the NIPPV effectively reduced the dyspnea score from 7.24 ± 1.58 at baseline to 5.53 ± 1.82 at one hour, to 4.11 ± 1.75 at six hours, 2.60 ± 1.03 at 24 hours, and 1.26 ± 0.44 at the time of discharge, respectively. The observed values were found to be significantly different when compared from baseline to all measurements (*P* < 0.0001). The RR decreased from 33.86 ± 4.98 minutes at baseline to 29.31 ± 5.81 minutes at one hour, to 24.23 ± 6.68 minutes at six hours, 20.57 ± 4.05 minutes at 24 hours and 16.76 ± 2.38 minutes during discharge. This was found to be significant (*P *< 0.0001) from baseline to all four measurements. The HR reduced from 105.42 ± 10.28 beats per minute (bpm) at baseline to 100.08 ± 10.61 bpm at one hour to 95.91 ± 9.95 bpm at six hours to 89.24 ± 9.98 bpm at 24 hours and 85.79 ± 7.98 bpm during discharge. This was found to be significant (*P *< 0.0001) from baseline to all four measurements. The data are shown in Table 2.

**Table 2:** Changes in the dyspnea score and hemodynamic parameters before, during, and after NIPPV. The comparison was done using a one-way analysis of variance (ANOVA). *Significant *P* < 0.0001 from the baseline. NIPPV, noninvasive positive pressure ventilation; bpm, beats per minute

Parameters	0 hours (*n* = 50)	1 hour (*n *= 49)	6 hours (*n *= 44)	24 hours (*n* = 42)	Discharge (*n* = 42)	*F*-value	*P*-value
Dyspnea score	7.24 ± 1.58	5.53 ± 1.82*	4.11± 1.75*	2.60± 1.03*	1.26± 0.44*	305.167	0.000*
Heart rate (bpm)	105.43 ± 10.28	100.28 ± 10.6*	96.71 ± 9.97*	90.49 ± 10.22*	86.68 ± 8.32*	70.536	0.000*
Respiratory rate (breaths per minute)	33.86 ± 4.98	29.31 ± 5.8*	24.23 ± 6.68*	20.57 ± 4.05*	16.76 ± 2.38*	227.360	0.000*

The pH changed from 7.24 ± 0.80 at baseline to 7.30 ± 0.07, 7.36 ± 0.06, 7.39 ± 0.03, and 7.41 ± 0.02 at one hour, six hours, 24 hours, and at discharge, respectively, and found to be significant when compared to baseline (*P *< 0.0001). Meanwhile, PaCO2 decreased significantly with time from 75.68 ± 15.14 to 67.61 ± 14.42, 60 ± 11.14, 52.31 ± 7.27, and 46.31 ± 6.21 at one hour, six hours, 24 hours, and at discharge, respectively, and found to be significant when compared to baseline (*P *< 0.0001). Further, an increase in PaO2 was observed from 57.4 ± 13.17 to 66.41 ± 12.84 at one hour, to 70.93± 14.57 at six hours, 71.6 ± 8.91 at 24 hours, and 74.40 ± 6.65 during discharge and found to be significant when compared to baseline (*P *< 0.0001). Results are shown in Table 3.

**Table 3:** Changes in pH and arterial blood gas parameters before, during, and after NIPPV. The comparison was done using a one-way analysis of variance (ANOVA). *Significant *P* < 0.0001 from baseline. NIPPV, noninvasive positive pressure ventilation; PaCO2, partial pressure of carbon dioxide; PaO2, partial pressure of oxygen

Parameters	0 hours (*n *= 50)	1 hour (*n *= 49)	6 hours (*n *= 44)	24 hours (*n *= 42)	Discharge (*n *= 42)	*F*-value	*P*-value
pH	7.24 ± 0.08	7.30 ± 0.08*	7.36 ± 0.065*	7.39 ± 0.036*	7.41 ± 0.023*	119.912	0.000*
PaCO2	75.68 ± 15.14	67.61 ± 14.42*	60 ± 11.14*	52.31 ± 7.27*	46.31 ± 6.21*	141.123	0.000*
PaO2	57.4 ± 13.17	66.41 ± 12.84*	70.93 ± 14.57*	71.6 ± 8.91*	74.40 ± 6.65*	24.976	0.000*

**Figure 1:**
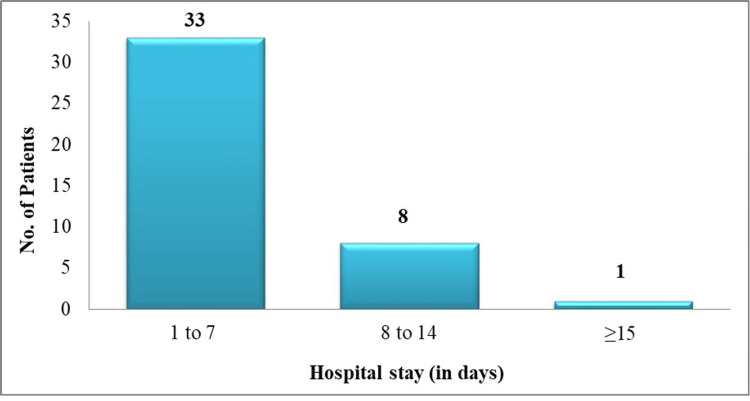
The duration of hospital stay for patients undergoing NIPPV. NIPPV, noninvasive positive pressure ventilation

In this study, the mean inspiratory positive airway pressure (IPAP) was found to be 16.90 ± 1.035 mmHg, and the mean expiratory positive airway pressure (EPAP) was 5.08 ± 0.444 mmHg. Further, the duration of NIPPV in this study was 25.70 ± 14.704 hours. The average length of hospitalization in the current study was 6.08 ± 3.029 days. The majority of patients were discharged within one to seven days (33 patients, 78.6%), followed by 8-14 days (eight patients, 19%), and more than 15 days (one patient, 2.4%). Results are shown in Figure 1.

In this study, 4 (14%) patients showed complications, and none of the patients needed discontinuation of ventilator support. Regarding complications, one (2%) patient had aspiration pneumonia and eye irritation, dryness of mouth in three (6%) patients, and pressure sores in two (4%) patients. 

The success rate of NIPPV in our observation was found to be 84%, and successful weaning was observed among 42 patients who were subsequently discharged. Among the eight NIPPV failures, two patients did not provide consent and left against medical advice. The remaining six patients were subjected to mechanical ventilation. Mortality was observed in four patients: two patients died due to ventilator-associated pneumonia (VAP), one patient due to gram-negative sepsis and multiple organ dysfunction syndrome (MODS), and the remaining patient due to a cerebrovascular accident.

## Discussion

Recently, the global incidence of COPD has increased, and it is one of the most frequent causes of mortality [10]. Restoring patients' respiratory function is the mainstay of COPD management in the presence of respiratory failure [11]. In the present scenario, mechanical ventilation is one of the preferred treatment modalities, and it is classified as invasive and noninvasive ventilation.

When compared to invasive ventilation, noninvasive ventilation also elicits ventilation, but with reduced ventilator-associated complications and a good prognostic value [12]. Earlier studies indicated a significant decrease in the need for endotracheal intubation and mechanical ventilation in the intensive care unit (ICU). Additionally, there is evidence of improved survival rates, reduced complication rates, and shorter stays in both the ICU and hospital [13,14].

NIPPV was successful among 84% of patients with acute exacerbation of COPD (AECOPD), as observed in our study. Similarly, a study in Egypt documented the success rate of NIPPV at 76% [15]. The result of the present study displayed a higher success rate as compared to the reported earlier studies, ranging between 50% and 80% [16,17]. In addition, NIPPV is effective, with a success rate of 89% in COPD subjects with respiratory acidosis with an arterial pH of 7.22 [18]. The diversity in success rates observed across various studies may be attributed to differences in patient characteristics and the severity of the diseases being studied.

In this current study, there was a substantial enhancement in the clinical ABG values following one hour of NIPPV application. In addition, there was a significant improvement in dyspnea score from 7.24 ± 1.58 at baseline to 5.53 ± 1.82 (*P *< 0.0001) at one hour. Further, there was a significant reduction in the respiratory rate from baseline of 33.86 ± 4.98 to 29.31 ± 5.81 (*P *< 0.0001). There was a substantial enhancement in the average PaCO2 levels within one hour, from 75.68 ± 15.14 to 67.61 ± 14.42. This change was also reflected in pH with improvements from 7.24 ± 0.08 to 7.30 ± 0.08. The PaO2 also changed from 57.40 ± 13.17 to 66.41 ± 12.48. Likewise, research reported by Khilnani et al. showed a marked increase in mean pH from a baseline of 7.23 ± 0.07 to 7.27 ± 0.08 (*P* < 0.001) [19]. Correspondingly, another study performed by Raju et al. documented a marked decrease in PaCO2 levels from baseline to after 5 hours: 54.90 ± 4.13 vs. 49.63 ± 2.87 (*P *< 0.001) [20]. Verma et al. documented a significant increase in PaO2 from baseline and 53.4 ± 18 to 71 ± 24.1 at discharge [21].

In our study, the duration of NIPPV was 25.70 ± 14.70 hours. Similarly, in a study done by Aggarwal et al., NIPPV was 19.6 hours [22]. The incidence of complications related to NIPPV complications was 14%. The major complications observed in the present study were NIPPV failure in 5.9% and pneumonia in 0.8% of the patients. In line with the present study, the previous studies reported the incidence of aspiration pneumonia as less than 5% [18,19,23]. In our study, the mean duration of hospital stay was found to be 6.08 days. However, a study documented the duration of hospital stay as 8.2 days, which was higher when compared to our study outcomes [24]. The shorter duration of hospitalization seen in this study may be attributed to the early restoration of blood gases, lack of sedation, reduced incidence of problems, and shorter weaning periods.

The limitation of our study is the small sample size due to a single-centered study, which limits its generalizability to a larger population. Additionally, NIPPV is restricted to patients with acute exacerbations of COPD. However, it is advisable to administer NIPPV before the onset of severe acidosis to prevent intubation and minimize consequences.

## Conclusions

According to the aforementioned results, our study shows that NIPPV is a potential therapeutic strategy for managing subjects with exacerbations of COPD who have developed respiratory acidosis after receiving routine medical treatment. Timely intervention with NIPPV might improve the symptoms and maintenance of normal blood gas variables and thus prevent intubation and invasive ventilation and their related complications.
